# Assessment of psychological factors affecting the functioning of patients undergoing bariatric surgery

**DOI:** 10.1192/j.eurpsy.2026.10765

**Published:** 2026-07-31

**Authors:** K. Rachubińska, K. Papierska, D. Schneider-Matyka, E. Grochans, M. Nowak, A. Cybulska

**Affiliations:** 1 Department of Nursing; 2 Students’ Scientific Association at the Department of Nursing, Pomeranian Medical University in Szczecin, Szczecin, Poland

## Abstract

**Introduction:**

Obesity is a chronic, multifactorial disease and a major public health challenge, characterized by excessive body fat accumulation leading to metabolic disturbances and comorbidities. Contemporary obesity management includes lifestyle modification, pharmacotherapy, and, in severe cases, bariatric surgery. Psychological factors, including body image perception, self-efficacy, self-esteem, and depressive symptoms, play a crucial role in treatment decisions and outcomes.

**Objectives:**

The study aimed to identify and analyze factors influencing body image perception among patients with obesity undergoing bariatric surgery.

**Methods:**

The study was conducted among 125 participants. Data were collected using a diagnostic survey that comprised a self-designed questionnaire alongside standardized instruments, including the Body Esteem Scale (BES), the General Self-Efficacy Scale (GSES), the Rosenberg Self-Esteem Scale (RSES), and the Beck Depression Inventory (BDI).

**Results:**

Motivations for bariatric surgery varied, with the majority aiming to improve overall wellbeing (49.6%) and achieve sustained weight loss (25.6%). Sociodemographic variables such as age, marital status, education, residence, and employment significantly influenced self-perception and psychological measures. Older age was associated with lower self-esteem, while individuals in informal relationships reported higher SES scores. Higher depressive symptoms were observed among married patients and those with a family history of obesity. Strong correlations were found between body image, self-efficacy, and self-esteem―higher self-efficacy and self-esteem were associated with better physical attractiveness, weight control, and physical fitness. Lower self-esteem and self-efficacy were linked to higher depression scores. In women, higher self-efficacy and self-esteem correlated with improved physical attractiveness and fitness perception; in men, higher self-esteem was associated with higher ratings of attractiveness, strength, and fitness.

**Conclusions:**

The patients undergoing bariatric surgery exhibited substantial body dissatisfaction, low self-efficacy, and low self-esteem, which were closely related to depressive symptoms. Sociodemographic and psychological factors significantly influenced body perception and treatment-related decisions. Family history of obesity increased the risk of depressive symptoms. These findings highlight the importance of integrating psychological assessment and support in bariatric surgery planning.

**Disclosure of Interest:**

None Declared

